# Infantile type I pleuropulmonary blastoma presenting with dyspnea due to compression by pneumothorax and an occupying tumor: a case report

**DOI:** 10.1186/s40792-023-01777-7

**Published:** 2023-11-06

**Authors:** Mitsumasa Okamoto, Shoka Kimura, Machiko Hotta, Yudai Tsuruno, Hiroaki Fukuzawa

**Affiliations:** 1 Department of Pediatric Surgery, Himeji Red Cross Hospital, 1-12-1, Shimoteno, Himeji, Hyogo 670-8540 Japan; 2 Department of Pathology, Himeji Red Cross Hospital, 1-12-1, Shimoteno, Himeji, Hyogo 670-8540 Japan

**Keywords:** Type I pleuropulmonary blastoma (PPB), Congenital pulmonary airway malformation (CPAM) type 4, Extrapulmonary cystic lung lesion, Tumor rupture, *DICER1* gene mutation

## Abstract

**Background:**

Pleuropulmonary blastoma (PPB) is an extremely rare and malignant pediatric lung tumor. Purely cystic PPB has a more favorable prognosis than solid PPB, but may be difficult to distinguish from a certain type of “benign” congenital pulmonary airway malformation before and during surgery. The influence of tumor rupture on long life prognosis has not been clarified in detail.

**Case presentation:**

A 5-month-old boy underwent emergency transfer from another hospital due to a left thoracic cystic lesion and left pneumothorax detected on chest radiography performed for persistent wheeze and cough. Contrast-enhanced computed tomography of the chest revealed marked deviation of the mediastinum to the right due to a giant cystic lesion and pneumothorax. Thoracotomy was performed on hospital day 2. A cystic lesion had developed from the distal alveolar region of lower lobe of the left lung and the tumor showed a tiny adhesion to the left diaphragm and a tiny rupture near the adhesion. Partial lung excision including the cyst and scraping of the adhesion were performed. Histopathological investigations revealed immature blast cell-like mesenchymal cells and differentiated striated muscle cells in a dense cambium layer were found under the epithelium of the cystic lesion. Type I PPB was diagnosed.

**Conclusions:**

Surgery should be performed with the possibility of type I PPB in mind when an extrapulmonary cystic lung lesion is found. Since issues such as the pathogenesis and long-term prognosis of ruptured cases remain unclear, continued careful follow-up of this case will be required.

## Background

Pleuropulmonary blastoma (PPB) is an extremely rare and malignant pediatric lung tumor [1]. The prognosis for type I (cystic lesion) PPB appears more favorable than that of type II (intermediate between type I and type III) or III (solid lesion) [2, 3], but differentiation from “benign” congenital pulmonary airway malformation (CPAM) by diagnostic imaging or from gross appearance of the lesion may be difficult [4]. Due to the small number of cases of type I PPB reported to date, the influence of tumor rupture on long life prognosis has yet to be elucidated in detail.

## Case presentation

**Fig. 1:**
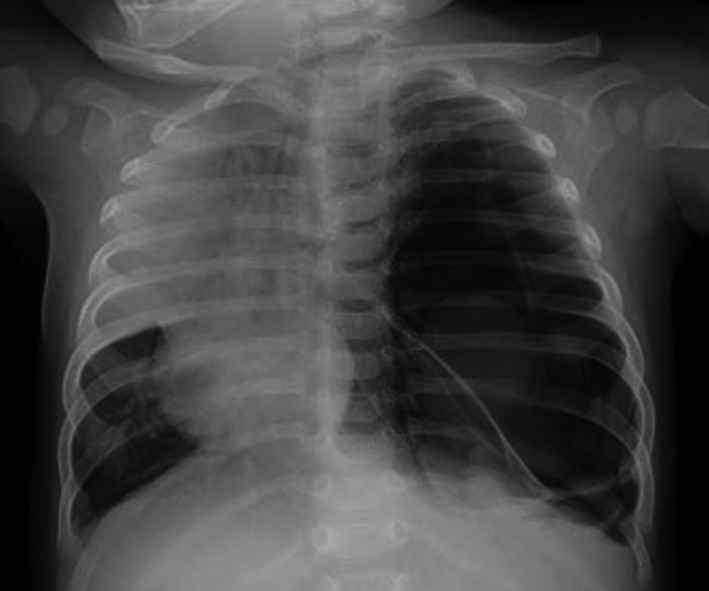
Chest X-ray on the day of transfer. A left thoracic cystic lesion and left pneumothorax are apparent

A 5-month-old boy with no other medical history underwent emergency transfer from another hospital due to a left thoracic cystic lesion and left pneumothorax detected on chest radiography, which had been taken because of a 2-week history of persistent wheeze and cough (Fig. 1).

**Fig. 2:**
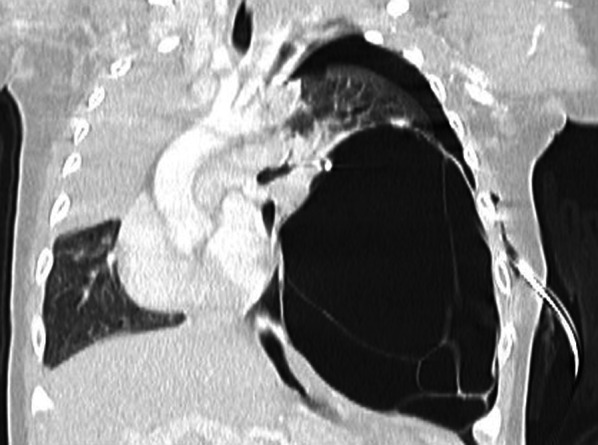
Contrast-enhanced computed tomography of the chest after insertion of a chest drainage tube. Severe mediastinal deviation to the right is evident, due to giant cystic lesion and pneumothorax

Chest contrast-enhanced computed tomography taken after insertion of a chest drainage tube revealed marked deviation of the mediastinum to the right (Fig. 2).

**Fig. 3:**
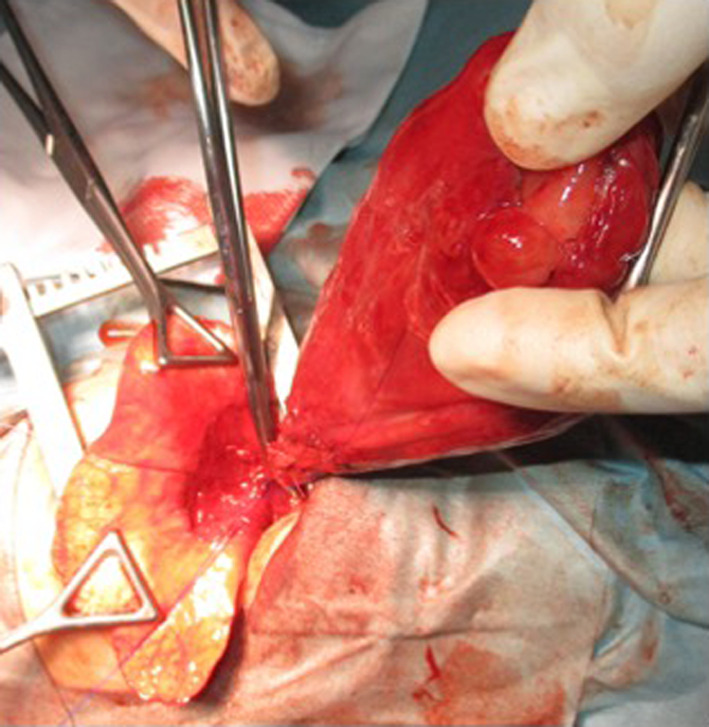
Operative findings. A cystic lesion is seen developing from the distal alveolar region of the lower lobe of the left lung

Surgery was performed on hospital day 2 because retractive breathing was unimproved with oxygen administration through a nasal cannula while under sedation with Dexmedetomidine. Thoracotomy was done by the left fifth postero-lateral intercostal incision in the lateral position under general anesthesia. A cystic lesion was seen to have developed from the distal alveolar region of the left lower lobe (Fig. 3). The tumor showed a tiny adhesion to the left diaphragm, with a tiny rupture near the adhesion. Since it was difficult to determine whether the lesion was benign or malignant during surgery, partial lung resection with cystic lesion and adhesion detachment were performed. Lymph node dissection was not performed in the surgery because there are currently no established clinical practice guidelines for PPB that can be used to predict disease prognosis or guide treatment like the Tumor, Node, Metastasis (TNM) staging system.

**Fig. 4:**
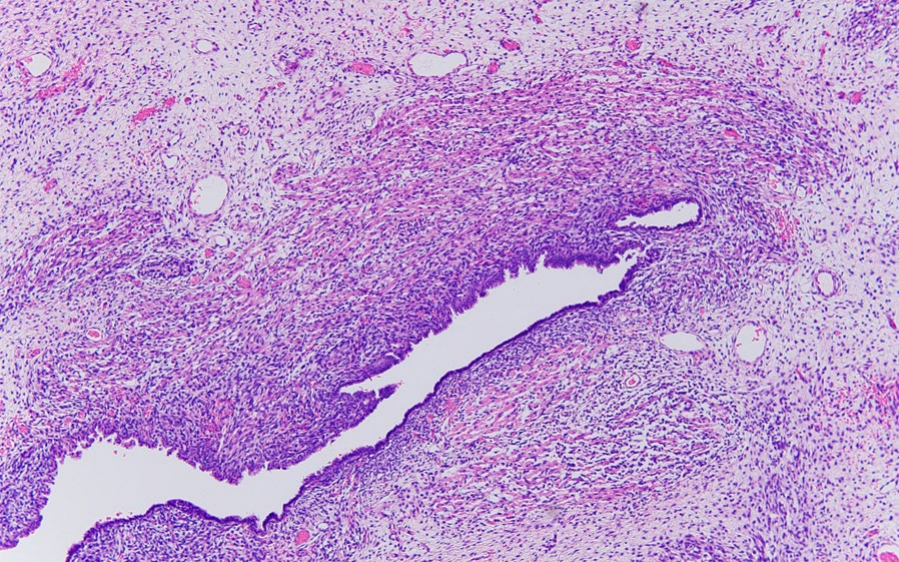
Pathological findings. Immature blast cell-like mesenchymal cells and differentiated striated muscle cells under the epithelium of the cystic lesion are observed. Hematoxylin and eosin, original magnification ×100

**Fig. 5:**
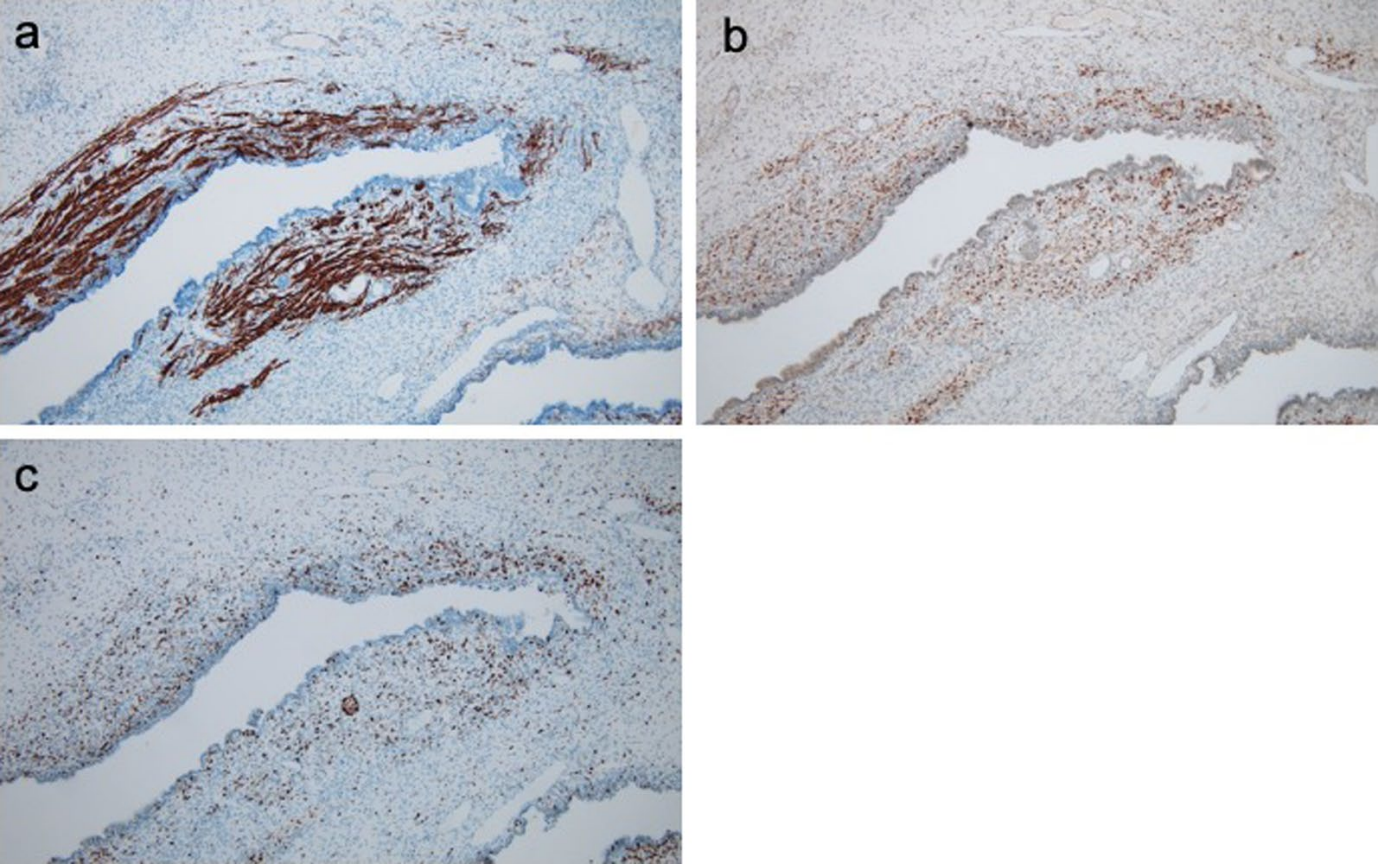
Immunohistochemical findings. Cells positive for desmin (**a**), myogenin (**b**) and Ki67 (**c**) are apparent in the tumor. Original magnification ×100

Histopathological investigations revealed immature blast cell-like mesenchymal cells and differentiated striated muscle cells present in a dense cambium layer under the epithelium of the cystic lesion (Fig. 4). Immunostaining of the excised specimen showed positive findings for desmin in immature mesenchymal cells and striated muscle differentiated cells. Striated muscle differentiated cells showed positive staining for myogenin. Ki67 staining showed a high positive rate (hot spot; 80%) consistent with immature mesenchymal cells showing a cambium layer-like growth pattern (Fig. 5). The patient was pathologically diagnosed as type I PPB.

Because the respiratory condition quickly improved and an atelectasis in the right upper lung field disappeared after surgery, the patient was transferred to another hospital and received 5 courses of VAC (vincristine, actinomycin D and cyclophosphamide) chemotherapy. About a year since surgical treatment and chemotherapy, the patient is doing well without recurrence.

Genetic testing revealed that both the patient and his mother had the hereditary mutation in *DICER1* gene.

## Discussion

PPB is a rare pediatric pulmonary malignancy [1]. In 1994, Dehner established the current disease concepts as: type I, purely cystic lesion; type II, intermediate between types I and III; and type III, predominantly solid lesion [2].

Type I PPB is understood to show better prognosis than type II or III [2, 3]. However, with or without chemotherapy, 10% of type I PPB cases may progress to type II or III after surgical resection [3].

The similarities of PPB and between CPAM have been discussed [4–6]. In 2002, Stocker proposed the new disease concept of CPAM, which was classified according to the site of lesion development in the tracheobronchial tree [6], from congenital cystic adenomatoid malformation (CCAM), which had been classified according to histomorphology [7]. In particular, CPAM type 4 is derived from distal acini and is itself uncommon (~ 10% of the CPAM cases) [6], so differentiation from cystic type I PPB based only on appearance may difficult. As a result, pathological diagnosis becomes extremely important [5].

The patient in this case presented with respiratory failure due to compression of the mediastinum by a space-occupying tumor rather than pneumothorax, so resection through thoracotomy was performed. Feinberg et al. proposed a therapeutic algorithm for cystic lung disease, suggesting that surgical treatment be performed if symptoms are present [8], while keeping malignancy in mind during those surgical procedures.

In this case, 5 courses of VAC chemotherapy were administered because rupture had occurred and tumor cells were also found at the diaphragm adhesion site. Pneumothorax due to tumor rupture is one of the problems with cystic PPB lesions [9, 10], but possibly due to the small number of cases reported to date, detailed comparison of long-term prognosis between ruptured and non-ruptured cases in type I PPB has been not described.

In 2009, *DICER1* gene was identified as the gene responsible for the onset of familial PPB [11]. Mutations in *DICER1* cause abnormalities in proteins that produce micro ribonucleic acids (RNAs) involved in cell proliferation, apoptosis, and cell differentiation, leading to the development of various malignant tumors such as PPB, rhabdomyosarcoma, cystic nephroma, and thyroid tumors. These related malignancies are collectively referred to as *DICER1* syndrome [12]. A potential for progression from CPAM to PPB has been reported [4, 13, 14] and the mutation of *DICER1* gene is speculated to be associated with malignant transformation, although no mechanisms have been elucidated [15, 16]. Mutations in *DICER1* gene have been confirmed in approximately 60% of PPB patients and the presence or absence of mutations may not affect differences in PPB disease type or survival rate [3]. However, careful follow-up is required because the patient has the mutation in *DICER1* gene.

## Conclusions

We should perform surgery with suspicion of malignant type I PPB when an extrapulmonary cystic lung lesion is found. Since problems such as the pathogenesis and long-term prognosis in ruptured cases remain unsolved clearly, further accumulation of surgical cases is required to better understand this disease in the future. Careful follow-up is required because the patient had ruptured lesion and has the mutation of *DICER1* gene.

## Data Availability

Not applicable.

## Abbreviations

*CCAM*: Congenital cystic adenomatoid malformation
*CPAM*: Congenital pulmonary airway malformation
*PPB*: Pleuropulmonary blastoma
*RNA*: Ribonucleic acid
*VAC*: Vincristine, actinomycin D and cyclophosphamide
*TNM*: Tumor, node, metastasis
